# Investigating the Relationship of *G-137C*, *C-607A*, and *A-1447G* Polymorphisms in the Promoter of *IL-18* and *CXCL10* Inflammatory Genes with Prostate Cancer in an Iranian Population

**DOI:** 10.1155/2024/3997576

**Published:** 2024-09-25

**Authors:** Nahid Ahmadi, Seyyed Amir Yasin Ahmadi, Abdolreza Kheirollahi, Farhad Shahsavar

**Affiliations:** ^1^ Student Research Committee Lorestan University of Medical Sciences, Khorramabad, Iran; ^2^ Preventive Medicine and Public Health Research Center Psychosocial Health Research Institute Iran University of Medical Sciences, Tehran, Iran; ^3^ Hepatitis Research Center School of Medicine Lorestan University of Medical Sciences, Khorramabad, Iran

## Abstract

**Introduction:**

Genetic and environmental factors are involved in prostate cancer. The current study was conducted to study the relationship between *G-137C*, *C-607A*, and *A-1447G* polymorphisms in the promoter of *IL-18* and *CXCL10* inflammatory genes with prostate cancer.

**Methods:**

As a genetic association study with a case-control design, the genomes of people living in Khorasan, Iran, were compared in two groups of cases and controls. The genotype of the *A-1447G* polymorphism present in the *CXCL10* gene promoter was investigated by the PCR-RFLP method. PCR-SSP was used to study the genotype of *G-137C* and *C-607A* polymorphisms present in the *IL-18* gene promoter. Odds ratio (OR) and 95% confidence interval (CI) were reported.

**Results:**

One mutant allele in *CXCL10 A-1447G* polymorphism (AG) increased the chance of cancer (OR = 4.902, 95% CI = 2.70–8.87) and two mutant alleles (GG) increased more (OR = 7.174, 95% CI = 2.48–20.68). One mutant allele in *IL-18 G-137C* polymorphism (CG) increased the chance of cancer (OR = 5.583, 95% CI = 3.04–10.22) and two mutant alleles (CC) increased more (OR = 9.571, 95% CI = 3.10–29.46). One mutant allele in *IL-18 C607A* polymorphism (CA) increased the chance of cancer (OR = 5.359, 95% CI = 2.95–9.70) and two mutant alleles (AA) increased more (OR = 7.083, 95% CI = 2.61–19.15) (*P* < 0.001).

**Conclusion:**

According to the results, the mutant alleles in polymorphisms *CXCL10 A-1447G*, *IL-18 G-137C*, and *IL-18 C-607A* alleles were associated with an increased chance of prostate cancer in this population.

## 1. Introduction

Prostate cancer is a common malignancy in which the primary cancer cell or maternal cancer cell grows in the prostate. It is one of the most prevalent cancers in men. Every year, above 670,000 men are diagnosed with this cancer worldwide. According to the latest estimates of global cancer incidence, prostate cancer is the second most common cancer among male population worldwide and the fifth leading cause of cancer-related death in men. Prostate cancer is the second most common cancer (after skin cancer) in developed countries and the second cancer causing mortality (after lung cancer) in males [1, 2]. One in six men will get this cancer. Prostate cancer ranks third among the most common cancers in Iranian men with 18.5 cases per 100,000 people [2, 3].

The etiology of the disease is related to lifestyle and genetic factors. According to various studies, various genetic factors have been effective in creating and increasing the risk factor for this disease, and the effects of changes in them on cancer have been proven [4–6]. Based on various studies, the effect of vitamin D receptor and immune cytokines such as *IL-1B*, *IL-6*, *IL-8*, and *IL-10* polymorphisms as risk factors for prostate cancer has been proven in various studies [5–7].

Considering the importance of this disease, it is very necessary to identify other factors and risk factors related to the development of this disease. CXCL10 (C-X-C motif chemokine 10) protein, also known as IP-10 (interferon gamma-induced protein 10), is a member of the CXC chemokine family that binds to the CXCR3 receptor to show its biological effect. CXCL10 is involved in the movement of white blood cells from the vessel wall to the tissue (chemotaxis), inducing apoptosis, regulating cell growth, and mediating angiogenic effects. CXCL10 is related to a wide range of human diseases, consisting of immunodeficiency, infectious diseases, chronic inflammation, tumor growth, metastasis, and dissemination. In addition, CXCL10 is known as a major biomarker that can reduce disease severity and may be used as a prognostic marker for many diseases [8–10].

Interleukin-18 (IL-18) has a similar structure with IL-1*β* and is a member of the interleukin-1 family. Many lymphoid and nonlymphoid cells express the IL-18 that plays important roles in inflammatory pathways. The biological function of IL-18 is mainly *via* inducing the secretion of interferon gamma-*γ* (IFN-*γ*) from T-helper lymphocytes. This cytokine correlates with IL-12, participates in T-helper (Th) 1 differentiation, and hence, is important in host defense against viruses, fungi, and intracellular bacteria. The evidence of recent studies shows that the participation of IL-18 in differentiation of Th2 and ultimately the production of IgE from B lymphocytes has provided new viewpoints into the dual effects of IL-18 in Th1 and Th2 inflammatory responses. The combination of IL-18 with IL-12 can activate cytotoxic T-cells (CTLs) and also natural killer (NK) cells for production of IFN-*γ* and thus can participate in tumor immunity [11–13]. These findings show that these two factors can be very effective in immunity against tumors [10, 11, 13].

Therefore, changes in the genomic level of these factors can be effective in the production rate as well as the response of the immune system against cancer cells. Considering that the effect of genetic changes of *CXCL10* and *IL-18* factors in the development of prostate cancer has not yet been clearly defined and there have not been many studies in this field in the world, and in addition, there is still no study in Iran to determine the relationship between polymorphisms These two genes have not been performed, so the main objective of this study was to investigate the relationship between *G-137C*, *C-607A*, and *A-1447G* polymorphisms in the promoter of inflammatory genes *IL-18* and *CXCL10* with prostate cancer. This study is conducted for the first time in Iran, and the results of this study can create a new understanding and insight regarding the role of these two factors in prostate cancer.

## 2. Materials and Methods

### 2.1. Study Design

This genetic association study was conducted in an Iranian population regarding strengthening the reporting of genetic association (STREGA) studies statement. This study was approved by the ethics committee of Lorestan University of Medical Sciences (IR.LUMS.REC.1398.145), and informed consent was taken from the participants.

### 2.2. Setting and Participants

Genomes of people living in Khorasan, Iran, were eligible to enter this study in two groups of cases (who had confirmed prostate cancer pathology) and controls (healthy people), from the molecular research laboratory of Qaen Faculty of Medical Sciences, regarding all the ethical points and observing all the necessary technical points. The samples were randomly obtained from the gene bank of the faculty. From the viewpoint of population stratification, the dominant ethnicities of this study were Fars (almost 95%), Kurds, and Turks, which constituted less than 5% of the people in this study. The proportions of these ethnicities were matched between the groups.

### 2.3. Laboratory Investigation

To determine the genotype of the *A-1447G* polymorphism present in the *CXCL10* gene promoter (*G* as the mutant allele), polymerase chain reaction with restriction fragment length polymorphism (PCR-RFLP) method (Kyratec Thermocycler, Australia) was used using primers and the use of the restriction enzyme SacI. Also, to determine the genotype of *G-137C* and *C-607A* polymorphisms present in the *IL-18* gene promoter (C and A as the mutant alleles), the polymerase chain reaction with sequence specific primer (PCR-SSP) method (Kyratec Thermocycler, Australia) was used using specific primers. All the samples were extracted using a DNA extraction kit (Yekta-Tajhiz Azma, Tehran, Iran) and stored in a freezer at −20°C. After preparing the equipment and preparing the genome, the optimum time and temperature for each PCR step were accurately identified. The genotyping of the CXCL10 A-1447G polymorphism was performed using PCR-RFLP. The PCR reaction mixture consisted of 1 *μ*L (10 *μ*M concentration) each of forward and reverse primers, 12.5 *μ*L of a premade PCR master mix (0.25 U/ul Taq DNA Polymerase, PCR Buffer, 0.4 mM dNTPs, MgCl2) (Yekta Tajhiz Azma, Tehran, Iran), 2 *μ*L of genomic DNA (50 ng), and nuclease-free water added to reach a final volume of 25 *μ*L. After several steps of trial and error, the PCR setup for the *A-1447G* polymorphism in the *CXCL10* gene promoter and the *G-137C* and *C-607A* polymorphisms in the *IL-18* gene promoter are shown in Table 1. In other words, the optimal PCR setup for the A-1447G polymorphism in the CXCL gene was established. This involves an initial denaturation at 94°C for 5 minutes, followed by 30 cycles of 30 seconds at 94°C, 30 seconds at 65°C, and 30 seconds at 72°C, concluding with a final extension at 72°C for 5 minutes. A total of 10 *μ*L of PCR products were subjected to restriction enzyme digestion using SacI. The reaction mixture comprised 2 *μ*L of 10X SacI buffer and 1 *μ*L of SacI enzyme (10 U/*μ*L) (Thermo Fisher Scientific, USA), and 16 *μ*L nuclease-free water was added to each sample. The reaction mixture was incubated at 37°C. A subsequent gel electrophoresis analysis assessed the digestion efficiency by comparing the banding patterns of digested and undigested PCR product.

**Figure 1:**
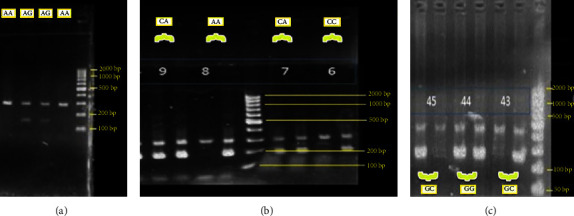
(a) Gel corresponding to the A-1447G polymorphism genotype present in the CXCL10 gene promoter. The type of the genotype and the size of the pieces are equal to AA: 290 bp. AG: 290 + 145 + 145 bp. GG: 145 + 145 bp; (b) gel corresponding to the C-607A polymorphism genotype present in the IL-18 gene promoter. The type of the genotype and the size of the parts are equal to 196 bp band which indicates the presence of G or C allele; 301 bp band for internal control; (c) gel related to G-137C polymorphism genotype present in IL-18 gene promoter. The type of the genotype and the size of the pieces are equal to band 261 bp which indicates the presence of G or C allele; 446 bp band for internal control.

To determine the genotype of the *A-1447G* polymorphism present in the *CXCL10* gene promoter, the RFLP-PCR method was used using specific primers and using the restriction enzyme SacI. After determining the setup tests to determine the genotype of *A-1447G* polymorphism present in the *CXCL10* gene promoter by the PCR-RFLP method and *G-137C* and *C-607A* polymorphisms present in the *IL-18* gene promoter by the PCR-SSP method for all samples were used in both cases and control groups. An SSP-PCR analysis of IL-18 polymorphisms was performed using a reaction mixture containing 0.8 *μ*L (10 *μ*M concentration) of forward primer (F1 or F2), 0.8 (10 *μ*M concentration) *μ*L of common reverse (CR) primer, 0.5 *μ*L (10 *μ*M concentration) of internal control (IC) primer, 2 *μ*L (50 ng) of DNA, 12.5 *μ*L of PCR master mix (0.25 U/ul Taq DNA Polymerase, PCR buffer, 0.4 mM dNTPs, MgCl2) (Yekta-Tajhiz Azma, Tehran, Iran), and nuclease-free water to reach a final volume of 25 *μ*L. In order to determine the genotype of the samples, the product obtained from PCR-SSP and PCR-RFLP was analyzed by running in 2% gel electrophoresis with Sifstein, and we see the result using the gel doc transilluminator (Poya Gostar, Iran). In order to prepare 2% agarose gel, we weigh the appropriate amount of agarose powder using a digital scale, and after dissolving it in TBE buffer, we put it on the heater and heat it. Close to the boiling point, put the gel under the hood and after it cools down a little, we add Sifstein to it and put the resulting suspension in the special casts of the gel and the combs of the gel. After preparing the gel, we pour the products with the loading buffer into the gel wells and place them in the electrophoresis tank with the negative direction towards the positive direction. Finally, after performing the electrophoresis, we can see the result using the gel doc transilluminator. Some examples of gel images are given in Figure 1.

### 2.4. Statistical Analysis

The sample size was calculated for the chi-square test of association between genotype exposure and cancer outcome. Considering 0.4 probability of exposure in the control group, 0.6 probability of exposure in the cancer group, two-tailed alpha 0.05, and 0.8 power, we needed at least 194 samples (97 individuals *per* group). However, 120 cases and 120 controls were considered. The chi-square test and the multiple logistic regression were to analyze the data. Odds ratio (OR) with 95% confidence interval (CI) were reported. Multiple comparison correction was not applicable as there was no hypothesis-free comparison. The area under the curve (AUC) was used to investigate the performance of logistic regression modeling. The Hardy–Weinberg equilibrium (HWE) was investigated using the chi-square test. SPSS 27 (IBM Corp., NY, US) was used for data analysis.

## 3. Results

### 3.1. Primary Findings

All the 240 participants had complete data. The average age of the studied samples was 64.17 ± 5.11 years, so that the lowest age was 55 years and the highest age was 73 years. The age was visually matched between the groups, but there was a not clinically important statistical difference (65.79 ± 4.67 years in cancer and 64.38 ± 5.01 years in the control group, *P*=0.024, independent *t*-test).

### 3.2. Genotype Distribution

For *CXCL10 A-1447G*, in 37.1% of samples both alleles were wild (AA), in 54.2% one allele (AG) and in 8.8% both alleles were mutant (GG). For *IL-18 G-137C*, in 36.7% of samples both alleles were wild (GG) and in 55% one allele (GC) and in 8.3% of cases, both alleles were mutant (CC). Also, for *IL-18 C607A*, in 39.2% of samples both alleles were wild (CC) and in 50.8% one allele (CA) and in 10% both alleles were mutant (AA) (Table 2). A deviation from HWE was observed for *CXCL10 A-1447G* and *IL-18 G-137C* polymorphisms (*P* < 0.05) (Table 2).

### 3.3. Association Study

**Figure 2:**
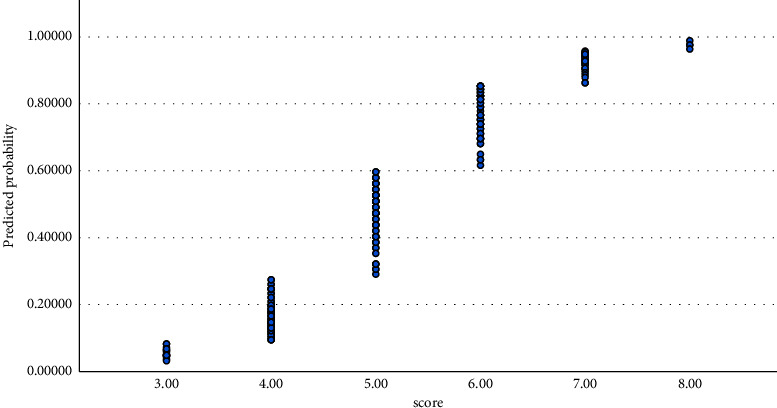
Sigmoid prediction of probability based on the genotype score for multiple logistic regression modeling of Table 3.

One mutant allele in *CXCL10 A-1447G* polymorphism increased the chance of cancer (OR = 4.902, 95% CI = 2.70–8.87, *P* < 0.001) and two mutant alleles increased more (OR = 7.174, 95% CI = 2.48–20.68, *P* < 0.001). One mutant allele in *IL-18 G-137C* polymorphism increased the chance of cancer (OR = 5.583, 95% CI = 3.04–10.22, *P* < 0.001) and two mutant alleles increased more (OR = 9.571, 95% CI = 3.10–29.46, *P* < 0.001). One mutant allele in *IL-18 C-607A* polymorphism increased the chance of cancer (OR = 5.359, 95% CI = 2.95–9.70, *P* < 0.001) and two mutant alleles increased more (OR = 7.083, 95% CI = 2.61–19.15, *P* < 0.001) (Table 2). In addition to these crude analyses, the effects of age and other polymorphisms of the study (as competing exposure) were adjusted (Table 2, Figure 2).

**Figure 3:**
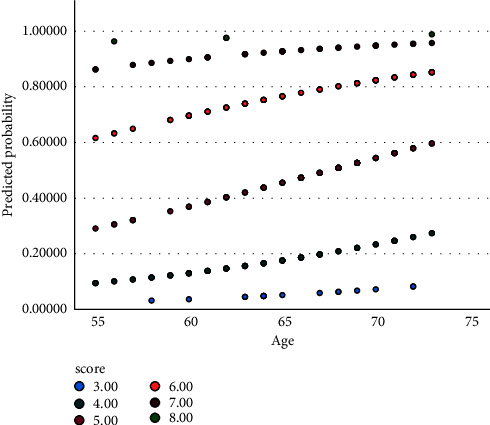
Sigmoid prediction of probability based on age divided by the genotype score for multiple logistic regression modeling of Table 3.

According to the results, a genotype scoring system was defined as the number mutant alleles for the three polymorphisms. Hence, each wild, heterozygote, and mutant genotype received one, two, and three scores, respectively. This scoring system showed a predictive role (OR = 3.915 per score, 95% CI = 2.75–5.57, *P* < 0.001) adjusted with age (Table 3, Figure 3).

## 4. Discussion

### 4.1. Interpretation

As the prostate cancer was the most common non-skin cancer and the second cause of death from malignancy in male population in developed countries [14], it was important to study the genetic aspects of this cancer. In this cancer, genetic changes may affect the clinical outcome [15]. According to the necessity of the investigation of reproducibility in genetic association studies, the present study was performed in an Iranian population. In other words, since ancestries might influence the results of genetic association studies, it was necessary to repeat each genetic association study in each specific population.

In the present study, the presence of one mutant allele in the *CXCL10 A-1447G* polymorphism significantly increased the chance of prostate cancer by 4.902 times and the presence of two mutant alleles significantly increased the chance of prostate cancer by 7.174 times. The presence of one mutant allele in the *IL-18 G-137C* polymorphism significantly increased the chance of prostate cancer by 5.583 times, and the presence of two mutant alleles significantly increased the chance of prostate cancer by 9.571 times. The presence of one mutant allele in the *IL-18 C607A* polymorphism significantly increased the chance of prostate cancer by 5.359 times, and the presence of two mutant alleles significantly increased the chance of prostate cancer by 7.083 times. These were large enough magnitudes of the effect in terms of effect size. Multiple logistic regression modeling approximately showed similar results demonstrating that age and the other investigated genotypes could not confound the directions of the effects in the present Iranian population. Finally, the present panel showed a good performance for the diagnosis of prostate cancer in this population (AUC = 0.863).

In a study performed by Abedinzadeh et al., the results showed that individuals having the *IL-18* mutant homozygous genotype (−607C > A and −137G > C) showed a higher risk of developing prostate cancer than controls [16], showing that these results were consistent with the results of the present study as both studies were on Iranian populations. It seems that prostate cancer is genetically predictable in Iranian populations. Also, Yuanyuan et al. showed that the *IL-18-607A* *>* *C* polymorphism decreased the risk of prostate cancer in an Asian population but increased the risk of prostate cancer in a Caucasian population [17]. Their results showed the role of ancestry in genetic disease association. Liu et al. investigated the role of *IL-18* polymorphisms (−607A > C and −137G > C) in susceptibility to prostate cancer in a Chinese population. They found −137 CC genotypes as a risk factor (OR = 2.18) as compared with the GG genotype. This finding was in line with the results of our study [18]. Regarding the *IL-18*-607C/A polymorphism, a meta-analysis was conducted by Yuanyuan et al. They found the CC genotype as compared with the AA genotype as a risk factor (pooled OR = 1.86) [17]. This finding was against the results in our study. It seems that the *IL-18*-607C/A polymorphism may have a different role in the Iranian population. Considering population stratification as a potential source of bias is important in genetic association studies.

Mulligan et al. stated that *CXCL10* acts on the tumor environment in a paracrine manner and on the tumor cells themselves in an autocrine manner and was involved in tumor invasion and progression [19] and showed that the results of this study could be aligned with the results of our study because we showed that *CXCL10* had an effect on the incidence of cancer. Jafarzadeh et al. showed higher levels of *CXCL10* in breast cancer patients which indicated that chemokines might play a role in tumor growth in breast cancer [8].

### 4.2. Limitations

The most important limitation of this study was lack of investigation-specific ethnic groups of Iran such as Kurd, Lur, and Azeri. However, the ethnicities of our samples were matched between the groups consisting of about 95% of Fars participants.

## 5. Conclusion

Based on the results of this study, polymorphisms in *CXCL10 A-1447G*, *IL-18 G-137C*, and *IL-18 C607A* alleles were associated with an increased risk of prostate cancer. The generalizability of this conclusion was limited to Fars or some mixed populations of Iran.

## Figures and Tables

**Table 1:** PCR setup for the *A-1447G* polymorphism in the *CXCL10* gene promoter and the *G-137C* and *C-607A* polymorphisms in the *IL-18* gene promoter.

Location	Genotype	PCR setup	Tm (°C)	Primers
*CXCL10–1447A* *>* *G*	AA: 290 bp AG:290 + 145 + 145bp GG: 145 + 145 bp	Begin denaturation at 94°C for 5 min, then perform 30 cycles of 30 s at 94°C, 30 s at 65°C, and 30 s at 72°C, ending with a 5 min extension at 72°C	65	F: 5′-TTGGTCAGGGAATGGAAAAG-3′ R: 5′-CGGTTTCCCACAGCTAATTC-3′

*IL-18-137G* *>* *C (rs187238)*	A 261 bp amplification product was observed for G and C and a 446 bp product for the control forward primer	Start denaturation at 94°C for 2 min, then proceed with 5 cycles of 20 s at 94°C, 1 min at 65°C, 1 min at 72°C, and 25 cycles at 94°C for 20 s, 61°C for 40 s, and 72°C for 40 s, concluding with a 5 min extension at 72°C	61	F1: 5′-CCCCAACTTTTACGGAAGAAAAG-3′ F2: 5′-CCCCAACTTTTACGGAAGAAAAC-3′ R: 5′-AGGAGGGCAAAATGCACTGG-3′ IC: 5′-CCAATAGGACTGATTATTCCGCA-3′

*IL-18-607 C* *>* *A*	A 196 bp amplification product was observed for C and A and a 301 bp product for the control forward primer	Begin denaturation at 94°C for 2 min, then conduct 7 cycles of 20 s at 94°C, 40 s at 64°C, 40 s at 72°C, and 25 cycles at 94°C for 20 s, 57°C for 40 s, and 72°C for 40 s, finishing with a 7 min extension at 72°C	57	F1: 5′-GTTGCAGAAAGTGTAAAAATTATTAC-3′ F2: 5′-GTTGCAGAAAGTGTAAAAATTATTAA-3′ R: 5′-TAACCTCATTCAGGACTTCC-3′ IC: 5′-CTTTGCTATCATTCCAGGAA-3′

IC: internal control.

**Table 2:** *CXCL A-1447G* polymorphism alleles compare between the two groups.

Polymorphism/Genotype	Control frequency (%)	Case frequency (%)	Crude OR (95% CI)^∗^	Adjusted OR (95% CI)^#^	Chi-square *P* value	HWE *P* value
CXCL10 A1447G					<0.001	0.022
Wild (AA)	66 (55)	23 (19.1)	Reference	Reference		
Heterozygous (AG)	48 (40)	82 (68.30)	4.902 (2.708–8.873)	6.118 (2.931–12.771)		
Mutant (GG)	6 (5)	15 (12.6)	7.174 (2.488–20.687)	7.441 (2.207–25.085)		
Total	120 (100)	120 (100)				
IL18 G-137C					<0.001	0.010
Wild (GG)	67 (55.8)	21 (17.5)	Reference	Reference		
Heterozygous (GC)	48 (40)	84 (70)	5.583 (3.049–10.224)	5.699 (2.776–11.701)		
Mutant (CC)	5 (4.1)	15 (12.5)	9.571 (3.109–29.469)	18.002 (4.212–76.946)		
Total	120 (100)	120 (100)				
IL18 C-607A					<0.001	0.227
Wild (CC)	70 (58.3)	24 (20)	Reference	Reference		
Heterozygous (CA)	43 (35.8)	79 (65.8)	5.359 (2.958–9.706)	7.935 (3.781–16.655)		
Mutant (AA)	7 (5.9)	17 (14.2)	7.083 (2.619–19.155)	5.588 (1.744–17.900)		
Total	120 (100)	120 (100)				

^∗^Simple logistic regression (*P* < 0.001). ^#^Multiple logistic regression, adjusted with other polymorphisms (*P* < 0.01) and age (OR = 1.066 per year, *P*=0.070), and AUC = 0.863.

**Table 3:** Logistic regression modeling for prediction of prostate cancer based on age and genotype score.

Variable	Adjusted OR (95% CI)	*P* value
Genotype score	3.915 (2.752–5.568)	<0.001
Age (year)	1.074 (1.004–1.148)	0.037
Constant	−11.633 (beta form)	<0.001

AUC = 0.847.

## Data Availability

The data used to support the findings of this study are available from the corresponding author upon reasonable request.
